# Gender-based differences in letters of recommendation written for ophthalmology residency applicants

**DOI:** 10.1186/s12909-019-1910-6

**Published:** 2019-12-30

**Authors:** Fei Lin, Soo Kyung Oh, Lynn K. Gordon, Stacy L. Pineles, Jamie B. Rosenberg, Irena Tsui

**Affiliations:** 1 Department of Ophthalmology, University of California, Los Angeles, CA USA; 2 Department of Ophthalmology and Visual Sciences, Montefiore Medical Center, Albert Einstein College of Medicine, Bronx, NY USA

**Keywords:** Ophthalmology, Residency, Gender, Application, Bias

## Abstract

**Background:**

To determine whether gender-based differences may be present in letters of recommendation written for ophthalmology residency applicants.

**Methods:**

All applications submitted through SF Match to the UCLA Stein Eye Institute Residency Training Program from the 2017–2018 application cycle were analyzed using validated text analysis software (Linguistic Inquiry and Word Count (Austin, TX)). The main outcome measures were differences in language use in letters of recommendation by gender of applicant.

**Results:**

Of 440 applicants, 254 (58%) were male and 186 (42%) were female. The two gender groups had similar United States Medical Licensing Exam (USMLE) Step 1 scores, undergraduate grade point averages (uGPA’s), proportions of underrepresented minority (URM) applicants and Gold Humanism Honor Society members, numbers of academic and service activities listed, and gender distributions of their letter writers (all *P* values > 0.05). However, letters written for male applicants were determined to use more “authentic” words than those written for female applicants (mean difference, 0.800; 95% CI, 0.001–1.590; *P* = 0.047). Letters written for male applicants also contained more “leisure” words (mean difference, 0.056; 95% CI, 0.008–0.104; *P* = 0.023) and fewer “feel” words (mean difference, 0.033; 95% CI, 0.001–0.065; *P* = 0.041) and “biological processes” words (mean difference, 0.157; 95% CI, 0.017–0.297; *P* = 0.028).

**Conclusions:**

There were gender differences detected in recommendation letters in ophthalmology consistent with prior studies from other fields. Awareness of these differences may improve residency selection processes.

## Background

In the past few years, ophthalmology residency selection has become a highly competitive process. The Ophthalmology Residency Match Summary Report 2017 revealed a mean United States Medical Licensing Examination (USMLE) Step 1 score of 243 (interquartile range, 236–252) for applicants who successfully matched into residency programs, compared to the United States and Canada mean (SD) of 229 (20). In 2017, 22% of ophthalmology residency applicants did not match into any program.

Competitive residency programs, including ophthalmology programs, rely heavily on cognitive measures in their selection processes to recruit top-tier medical school graduates [1]. However, cognitive metrics, including USMLE Step 1 scores, have been poor predictors of success in residency. In 2002, the Accreditation Council for Graduate Medical Education identified six Core Competencies that residents must master in their training. Imbedded in those competencies are many non-cognitive factors, such as interpersonal and communication skills, which can be evaluated through letters of recommendation.

Thought to provide insight into applicants’ non-cognitive traits, letters of recommendation written on behalf of applicants can grasp the attention of selection committees. An online survey completed by program directors, chairpersons, and members of resident selection committees from 65 United States ophthalmology residency programs showed that 83% of programs considered letters of recommendation to be among the most important component of applications [2]. It has also been demonstrated that letters of recommendation were the most important factor for screening and evaluating applicants for medical schools, graduate programs, psychology faculty positions, internships, and military training programs [3–7]. Selection committees rely on letters of recommendation to provide information on applicants’ motivations, qualifications, and past performances, and to reinforce the information provided by the applicants themselves [7–9].

Recent research has demonstrated gender biases in letters of recommendation in medical specialties, including diagnostic radiology, emergency medicine, urology, and general and transplant surgery [10–14]. These studies used text analysis software programs, such as Linguistic Inquiry and Word Count (Austin, TX), and determined that letter writers tended to use different words to describe male and female applicants. Gender biases may be especially relevant in the field of ophthalmology, as women accounted for only 44% of ophthalmology residents in 2014 and 23% of the total number of practicing ophthalmologists in 2015 [15]. The purpose of this study is to explore gender-based differences in recommendation letters written for ophthalmology residency applicants.

## Methods

This study was exempt from review by the Institutional Review Board because only de-identified data were used for analysis. All 440 residency applications submitted through SF Match to the UCLA Stein Eye Institute Residency Training Program from the 2017–2018 application cycle were included in this study. Each application was reviewed to extract demographic information, namely gender and self-reported underrepresented minority (URM) status. The Public Services and Activities section of each application was reviewed to count the number of academic and service activities listed. Academic activities included tutoring, mentoring, and research-related activities. Service activities included volunteering in clinics and working on community projects. Academic achievement data collected included USMLE Step 1 scores and undergraduate grade point averages (uGPAs). Finally, Gold Humanism Honor Society member status in medical school was noted, and the gender of the letter writer for each letter of recommendation was documented.

Letters of recommendation were prepared for analysis by using Adobe Acrobat Pro™ (San Jose, CA) to remove all headings, greetings, and signatures. The latest (2015) version of Linguistic Inquiry and Word Count (LIWC) was used to analyze the main text of each letter. For each letter, LIWC2015 outputted a number for each word category, with larger values corresponding to increased use of words in that particular category.

LIWC has been validated and used in studies in other scientific fields to detect gender biases in letters of recommendation, as well as to predict numerous outcome measurements, including social judgments, psychological adjustments, personality changes, personalities, and health [10–14, 16–22]. LIWC2015 contains nearly 90 output variables, including 4 summary language variables (analytical thinking, clout, authenticity, and emotional tone), 41 categories related to psychological constructs (including affect, cognition, biological processes, and drives), and 6 categories related to personal concerns (including work, home, and leisure activities). LIWC2015 reads each word of the text being analyzed and increments the output variable(s) that contain(s) the word. For example, the word “cried” is included in 5 output variables: sadness, negative emotion, overall affect, verbs, and past variable. Nearly 6400 words and word stems are included in LIWC2015, and each word or word stem was placed under the appropriate output variable(s).

The results of the analyses performed by LIWC2015 were collected in a Microsoft Excel spreadsheet (Redmond, WA) and subsequently analyzed using Stata/MP 15 (College Station, TX). For each LIWC2015 output category, a two-sample, two-tailed t test was performed with respect to applicant gender under the settings of unpaired data, unequal variances, and Welch’s approximation.

## Results

A total of 440 applicants (254 males and 186 females) and 1318 letters of recommendation were included in this study.

There were no differences between male and female applicants in USMLE Step 1 scores (mean difference, 1.82; 95% CI, − 4.46 to 0.830; *P* = 0.18), UGPAs (mean difference, 0.014; 95% CI, − 0.058 to 0.030; *P* = 0.54), proportion of URM applicants (mean difference, 0.053; 95% CI, − 0.008 to 0.114; *P* = 0.087), proportion of Gold Humanism Honor Society members (mean difference, 0.027; 95% CI, − 0.080 to 0.026; *P* = 0.32), number of academic activities (mean difference, 0.276; 95% CI, − 0.006 to 0.558; *P* = 0.055), number of service activities (mean difference, 0.373; 95% CI, − 0.162 to 0.908; *P* = 0.24), and gender distribution of their letter writers (mean difference, 0.024; 95% CI, − 0.079 to 0.031; *P* = 0.25). Table 1 summarizes these results. Due to lack of differences in application profiles between male and female applicants, subgroup analyses of above parameters were not pursued. Subgroup analysis of the letters with respect to URM status was not pursued because URM applicants had lower USMLE Step 1 scores (mean difference, 12.35; 95% CI, 7.06 to 17.65; *P* < 0.001) and UGPAs (mean difference, 0.153; 95% CI, 0.061 to 0.246; *P* = 0.002) compared to non-URM applicants, and these differences would be potential confounders.

**Table 1:** Comparison of the 254 male and 186 female applicants to the UCLA Stein Eye Institute Residency Training Program from the 2017–2018 application cycle

Measure	Male Applicants, Mean (SD)	Female Applicants, Mean (SD)	Difference in Means (95% CI)	*P* Value
USMLE Step 1 score	244.7 (13.61)	242.89 (14.11)	1.82 (−4.46 to 0.830)	0.18
UGPA	3.74 (0.23)	3.76 (0.22)	0.014 (−0.058 to 0.030)	0.54
Proportion of URM applicants	0.087 (0.282)	0.140 (0.348)	0.053 (−0.008 to 0.114)	0.087
Proportion of Gold Humanism Honor Society members	0.102 (0.304)	0.075 (0.264)	0.027 (−0.080 to 0.026)	0.32
Number of academic activities	1.24 (1.48)	1.52 (1.49)	0.276 (−0.006 to 0.558)	0.055
Number of service activities	5.20 (2.94)	5.57 (2.74)	0.373 (−0.162 to 0.908)	0.24
Proportion of female letter writers	0.725 (0.489)	0.701 (0.514)	0.024 (−0.079 to 0.031)	0.25

Letters of recommendation written for male applicants were determined to be more “authentic” than those written for female applicants, with a mean difference of 0.800 (95% CI, 0.001–1.590; *P* = 0.047). Letters written for male applicants also contained more “leisure” words (such as “cook,” “chat,” and “movie”) than those written for female applicants, with a mean difference of 0.056 (95% CI, 0.008–0.104; *P* = 0.023). Finally, letters written for female applicants contained more “feel” words (such as “feels” and “touch”) and “biological processes” words (such as “eat” and “pain”) than those written for male applicants, with mean differences of 0.033 and 0.157, respectively (95% CIs, 0.001–0.065 and 0.017–0.297; *P* = 0.041 and 0.028, respectively). Table 2 summarizes the results of these four LIWC2015 output variables with gender-based differences.

**Table 2:** Differences in letters of recommendation written for the 254 male and 186 female applicants to the UCLA Stein Eye Institute Residency Training Program from the 2017–2018 application cycle

LIWC Output Variable	Male Applicants, Mean (SD)	Female Applicants, Mean (SD)	Difference in Means (95% CI)	*P* Value
Authentic	8.46 (7.53)	7.66 (7.00)	0.800 (0.001–1.590)	0.047
Leisure	0.48 (0.45)	0.42 (0.43)	0.056 (0.008–0.104)	0.023
Feel	0.28 (0.28)	0.31 (0.30)	0.033 (0.001–0.065)	0.041
Biological Processes	3.29 (1.21)	3.45 (1.33)	0.157 (0.017–0.297)	0.028

## Discussion

This study used validated text analysis software to analyze a large volume of recommendation letters written for ophthalmology residency applicants. We sought to determine if applicant gender influenced letter writers’ language use. Male and female applicants had no significant differences in USMLE Step 1 scores, uGPAs, proportions of URM applicants and Gold Humanism Honor Society members, numbers of academic and service activities listed, and gender distributions of their letter writers. These findings suggest that male and female applicants had achieved similar levels of success in both the academic and service settings, and applied for residency under similar circumstances. However, analysis of recommendation letters in our study revealed differences between letters written for male applicants and those written for female applicants in the following LIWC2015 output variables: authentic, leisure, feel, and biological processes.

In our study, letters of recommendation written for male ophthalmology residency applicants were determined, based on language style and use, to be more “authentic” than those written for female applicants. This result was also found in a recent study on gender biases in letters of recommendation written for urology residency applicants [10]. Creation of the “authentic” variable in LIWC2015 was inspired by literature on “reality monitoring” that suggested that stories based on real experiences are told and written in a qualitatively different manner from those that are falsified [23–25]. Specifically, true stories were more likely to be characterized by complexity and positivity, therefore implying a possible advantage for male applicants over female applicants.

Second, in our study, letters written for female applicants contained fewer “leisure” words than those written for male applicants, which may be a commentary on work ethic consistent with previous research suggesting that recommenders tend to emphasize women’s work ethic rather than ability or talent [26]. A past study on letters of recommendation written for successful applicants to faculty positions at a large medical school in the United States found that letters written for female applicants contained more grindstone adjectives, such as “committed” and “tireless,” than those written for male applicants. Furthermore, the study reported that compared to their male counterparts, female applicants were described with fewer adjectives that suggest ability and talent, such as “analytical” and “genius.” Another study on letters of recommendation written for applicants to chemistry and biochemistry faculty positions at a large research university in the United States found that letters of recommendation written for female applicants contained fewer standout adjectives, such as “magnificent,” “wonderful, and “superb,” than those written for male applicants [18].

Finally, letters written for female applicants contained more “feel” and “biological processes” words than those written for male applicants. Examples of “feel” words are “feels” and “touch,” and examples of “biological processes” words are “eat” and “pain.” It is uncertain what impact this may have on residency selection processes, but gender stereotypes that arose from traditional gender-based divisions of social roles may have contributed to these categorical word usage differences [27].

The main limitation to this study is that although LIWC2015 provided an objective measure of differential language use in the letters of recommendation, it cannot take context into account. Therefore, no conclusions other than differences in word selection can be made from the programmed analysis of the letters. Another limitation is that the letters were not interpreted by program directors, which may be more practical; however, that analysis would be non-objective and infeasible given the large number of ophthalmology residency applicants and letters. Future studies could examine the context in which the words were used and present results to program directors for interpretation on how the differences in word usage may impact how applicants are viewed and ranked by residency selection committees.

## Conclusions

In conclusion, although similar levels of success were achieved in both academic and service settings by male and female ophthalmology residency applicants, their letters of recommendation differed in word usage. The differences were consistent with gender observations previously reported in medicine and other scientific fields. Awareness of areas of potential gender biases is an important first step towards better selection processes in which decisions are made based on applicants’ achievements and qualifications.

## Data Availability

The datasets used and/or analyzed during the current study are available from the corresponding author on reasonable request.

## Abbreviations

LIWC: Linguistic Inquiry and Word Count
uGPA: undergraduate grade point average
URM: Underrepresented minority
USMLE: United States Medical Licensing Exam
